# Deletion of the benzoxazinoid detoxification gene *NAT1* in *Fusarium graminearum* reduces deoxynivalenol in spring wheat

**DOI:** 10.1371/journal.pone.0214230

**Published:** 2019-07-12

**Authors:** Thomas Baldwin, Suzette Baldwin, Kathy Klos, Phil Bregitzer, Juliet Marshall

**Affiliations:** 1 National Small Grains Germplasm Research Facility, USDA-ARS, Aberdeen, Idaho, United States of America; 2 Department of Plant, Soil, and Entomological Sciences University of Idaho Research and Extension, Idaho Falls, ID, United States of America; Seoul National University, REPUBLIC OF KOREA

## Abstract

Benzoxazinoid (Bx) metabolites produced by wheat and other members of the *Poaceae* have activity against *Fusarium sp*. that cause cereal diseases including Fusarium head blight (FHB) on wheat and barley. Certain Bx metabolites can be detoxified by *Fusarium* sp. with the arylamine N-acetyltransferase *NAT1*. Investigation of this pathway may reveal strategies for increasing FHB resistance, such as selection for higher levels of Bx metabolites within existing germplasm and/or engineering fungal susceptibility via host induced silencing of *NAT1*. We assessed the reactions of fifteen wheat cultivars or breeding lines adapted to the Northwestern United States to infection with *F*. *graminearum Δnat1* mutants that should be sensitive to Bx metabolites. Significant differences were noted in disease severity and deoxynivalenol (DON) among the cultivars 21 d after inoculation with either mutant or wildtype (PH1) strains. Mutant vs. wildtype strains did not result in significant variation for infection severity (as measured by % infected florets), but inoculation with *Δnat1* mutants vs. wildtype resulted in significantly lower DON concentrations in mature kernels (*p* < 0.0001). Of the cultivars tested, HRS3419 was the most resistant cultivar to PH1 (severity = 62%, DON = 45 ppm) and *Δnat1* mutants (severity = 61%, DON = 30 ppm). The cultivar most susceptible to infection was Kelse with PH1 (severity = 100%, DON = 292 ppm) and *Δnat1* mutants (severity = 100%, DON = 158 ppm). We hypothesized that sub-lethal Bx metabolite levels may suppress DON production in *F*. *graminearum Δnat1* mutants. *In vitro* assays of Bx metabolites BOA, MBOA, and DIMBOA at 30 μM did not affect growth, but did reduce DON production by *Δnat1* and PH1. Although the levels of Bx metabolites are likely too low in the wheat cultivars we tested to suppress FHB, higher levels of Bx metabolites may contribute towards reductions in DON and FHB.

## Introduction

Fusarium Head Blight (FHB) of wheat and barley in the United States is caused primarily by *Fusarium graminearum*, but also by *F*. *culmorum* and *F*. *pseudograminearum*. This devastating disease has the potential to reduce crop yields and quality in just a few weeks before harvest [1,2]. Efforts to breed resistance to this disease have resulted in modest improvements in both wheat and barley [3–5]. However, confounding factors such as genotype by environment interactions, markers co-segregating with agronomic traits, and broad resistance involving many gene pathways presents a significant challenge to breeding desired levels of resistance to FHB [6]. One strategy that could improve outlook of resistance breeding is by targeting host metabolites with known activity against the pathogen. This reverse genetics approach to breed host resistance will aid in selection of more resistant cultivars. Several candidate secondary metabolites with activity against *Fusarium sp*. that are produced in the Poaceae that have been correlated to reduced disease in the field [7] include gramine, linoleic acid, phenylalanine, carotenoids, phenolic acids, cinnamic acids, and benzoxazinoids. Benzoxazinoids, aka Bx metabolites, have been extensively studied with respect to their effects on *Fusarium sp*. [8–15].

Benzoaxazanoids (aka Bx metabolites) are known to have allelopathic effects on insects and fungi. Physical damage, herbivory, and infection by pathogens are known to trigger expression of Bx metabolites, activating a protective effect in the plant [16–20]. These effects can be indirect activation of plant biotic and abiotic stress responses, including callose formation [21], or by direct anti-herbivory antimicrobial properties of the metabolites. Allelopathy against the European corn borer on maize [22, 23], aphids in wheat and maize [21, 24, 25], and nematodes in rye [26, 27] is well documented. Inhibition of proteases and thus interference with insect digestion is one mechanism proposed by which Bx metabolites deter herbivory [25, 28]. Additionally, Bx compounds–natural and synthetic–have a wide range of antimicrobial activity in phytopathogenic species including, *Fusarium*, *Phythophtora*, *Rhizoctonia*, *Phoma*, *Alternaria*, *Blumeria*, and *Botytus* [9–13, 16, and 29].

Bx metabolites have been categorized as hydroxamic acids, lactams, benzoxazolinones, and methyl derivatives with different microbial properties [16, 17]. Bx metabolites can be glycosylated during biosynthesis and glycosides are unable to undergo ring-opening, making them less reactive [reviewed in 16].The profile of Bx metabolites are different among cereal species. Maize and wheat primarily produce DIMBOA, whereas rye and wild barley (*Hordeum lechleri*) primarily produce DIBOA [17, 30–32]. However, Bx metabolites are absent in other cereals, such as cultivated barley, *H*. *vulgare* [reviewed in 32]. In wheat, Bx metabolites are produced by Bx genes *TaBx1─5* [17]. *TaBx1─2* are on located on chromosome 4 and *TaBx3─5* are located on chromosome 5 [17, 33, 34]. Nomura et al., [34] suggested the B genome of wheat contributes the most to the expression of Bx genes. These genes encode the P450 proteins within the CYP71C subfamily that are responsible for the biosynthesis of the cyclic hydroxamic acids 2,4-dihydroxy-1,4-benzoxazin-3-one (DIBOA) and 2,4-dihydroxy-7-methoxy-1,4-benzoxazin-3-one (DIMBOA) from the primary metabolite indole-3-phosphate [17]. Interestingly, cultivated barley produces a related indole alkaloid from indole-3-phosphate, gramine, which has been implicated as allelopathic to pests and pathogens.

*Fusarium* species have a high capacity to detoxify Bx metabolites. The first studies of the effects of Bx metabolites on *Fusarium* was performed by Glenn et al. [9, 10], who found that the genetic clusters *FDB1* and *FDB2* in *F*. *verticillioides* are necessary for detoxifying the Bx metabolites MBOA and BOA. Recent analysis suggests that these clusters are a result of horizontal transfer events from *Colletotrichum* and *Aspergillus*, respectively [15]. One of the genes within the *FDB2* locus is an arylamine N-acetyltransferase, designated *NAT1*, which converts the intermediate degradation product of BOA, 2-aminopheno (2-AP), to the nontoxic N-(2-hydroxyphenyl) malonamic acid (HPMA) [10–12]. This conversion requires N-malonyltransferease activity specific to NAT1 of the NAT family, using malonyl-CoA as a substrate [15]. Deletion of *NAT1* from the *FDB2* cluster resulted in an inability of *F*. *verticillioides* to detoxify Bx metabolites [12]. When the *NAT1* homolog in *F*. *pseudograminearum* (referred to as *FDB2* by the authors) was deleted, the fungus could no longer grow on Bx metabolite-amended media [13]. Thus, *NAT1* is the gene within the *FDB2* locus responsible for Bx metabolite detoxification.

Detoxification of Bx metabolites may be important for virulence with respect to FHB. *F*. *pseudograminearum Δnat1 mutants* exhibited a decrease in virulence when inoculated on spikes of the wheat cultivar, Kennedy, presumably caused by Bx metabolites produced by the plant [13]. Consistent with this report, these authors [7, 35] reported a correlation of Bx metabolites to decreases in FHB and mycotoxin accumulation. In addition, Etzerodt et al., [7] found DIMBOA-Glu in wheat spikes from the field to be less than 20 μg/g (dry weight) in most winter and spring wheats cultivars tested.

These reports merit further investigation of the potential for Bx metabolites to contribute to FHB resistance in wheat on FHB caused by *F*. *graminearum*. We tested the effects of *Δnat1* mutants on deoxynivalenol (DON) production and the ability of mutants to tolerate the Bx metabolites DIMBOA, MBOA, and BOA. These mutants were then used to determine potential variability in Bx metabolite content among fifteen wheat cultivars and breeding lines adapted in the Northwestern United States. Barley was included as a non-Bx metabolite-containing control.

## Materials and methods

### Strains and growth conditions

The wild type *F*. *graminearum* strain PH1 (NRRL 31084, FGSC 9075) was used as a control and background strain for transformation. All strains were grown and maintained on V8 medium (see [36]) in 100 x 15-mm Petri plates. Mycelial growth assays and observations of mutants were performed on potato dextrose agar (Sigma-Aldrich, St. Louis, MO). For the production of conidia, agar plugs of 3-d-old cultures on V8 media were taken from actively growing mycelia along the periphery of the cultures and used to inoculate 100 mL of carboxymethyl cellulose (CMC) medium [36] in 250 mL-Erlenmeyer flasks, and incubated for 4–5 d at 25°C on a rotary shaker at 150 rpm. Conidia were isolated by centrifugation at 2500 x *g*, followed by resuspension in 1 mL of sterile, deionized H_2_O. Spore concentrations were determined using a hemocytometer.

### Deletion of *NAT1* in *F*. *graminearum*

A plasmid designed to introduce hygromycin resistance in place of *NAT1* via homologous recombination was created using the IIP plasmid [37] as the backbone as follows. Approximately 900 bp of the upstream and downstream regions of *NAT1* were PCR amplified with 20 bp overhangs as necessary for Gibson assembly [38] and assembled onto both sides of the hygromycin resistance cassette in IIP. PEG-mediated transformation of protoplasts was performed as described in [36] and the primers used to determine the correct insertion are listed in S1 Table. The restriction enzyme I-*Sce*I was used to linearize the plasmid for homologous recombination [39]. The *Δnat1* mutants have been recorded and made available via FgMutantDb [40]

### Virulence assays

Virulence on wheat was determined in three experiments. Wheat cultivars were planted in “Cone-tainers” (4-cm diameter × 20.5-cm long; Stuewe & Sons, Tangent, OR, USA) filled with a vermiculite/peat moss/sand mixture in a 1:1:1 ratio by volume and Osmocote 15-9-12 slow release fertilizer (25g/L; (Everris International, Dublin OH USA). Plants were grown in growth chambers under fluorescent and incandescent lights with a 16-h photoperiod at temperatures of 14°C (dark period) and 4 h at 16°C, 8 h at 20°C, and 4 h at 16°C (light period, 54─90 μmol m^-2^). Inoculation was performed as described in [36]. In brief, for each mutant strain, 10 μL of a solution composed of 5 x 10^5^ conidia/mL was injected with a syringe and needle into a single floret in each of three spikes per cultivar just prior to anthesis. The florets that were inoculated were 4─5 florets from the base of each spike. The spike was covered with a plastic bag for 3 d which was subsequently replaced with a glassine bag (2” x 8.5”; L404, Lawson Northfield, IL) Wheat spikes were harvested 21 dpi, dried in a paper bag for 3 d at 37°C, and pulverized to a powder for DON analysis using a SPEX Freezer/Mill® (Spex Industries Inc. Metuchen, NJ) as described above. For each experiment, spikes within treatments were combined.

Virulence on barley was determined in a single experiment. Barley, cultivar Golden Promise, was grown as described for wheat, except that for the first 7 wk the plants were grown in a greenhouse with diurnal temperatures from 12─25°C and a ~14 h photoperiod (artificial light added when natural light fell below 40 μmol m^-2^ s^-1^). Inoculation of barley heads was performed as described in [37]. Seven spikes per fungal strain were dip-inoculated. A mock inoculation was included as a control. After 21 d, infected florets were pulverized to a powder using a SPEX freezer mill as described above, and used for DON analysis.

### GC-MS DON analysis

DON analysis was performed at North Dakota State University, Institute of Barley and Malt Sciences. Analyses were as described in [37] In brief, either 500 mg or 1 g of pulverized infected tissues was extracted with 10 mL of 84% acetonitrile 16% water mixture, filtered, and diluted for sample analysis on a GC-MS. Concentration was adjusted per 1 g of sample.

### Bx metabolite plate assays

*F*. *graminearum* PH1 and *Δnat1* were exposed to 30 μM of BOA (157058 –Sigma-Aldrich, St. Louis, MO, USA), MBOA (711594 –Sigma-Aldrich), and DIMBOA (D459950—Toronto Research Chemicals, Ontario Canada), combined in 4 mL of media and inoculated at 10^5^ spores per mL. Samples were taken at 96 h and measured for growth (OD_600_) on Biotek Synergy HT spectrophotometer (Biotek, Winooski, VT, USA). Samples were freeze dried for DON analysis. Exposure of *F*. *graminearum* strain TRI5prom::GFP [41] to BOA, MBOA, and gramine (G10806—Sigma-Aldrich) at 3 mM was performed in 96 well plate assays. A Biotek Synergy HT spectrophotometer (Biotek) was used to measure growth (RFP) and GFP fluorescence.

### Statistical analysis

For severity and DON data comparison between cultivars and strains a statistical fixed effects ANOVA model was applied. To identify significant sources of variation between experiments, cultivar, and strain (PH1 vs. *Δnat1*) and varieties for both severity and DON, F values were calculated for treatment effects and compared to the probability function to determine significance level. Models were derived using SAS software. Significance was determined at *p =* 0.05).

## Results

### Identification and deletion of *F*. *graminearum NAT1*, and characterization *of Δnat1 mutants*

There are three *NAT* orthologs in *F*. *graminearum*. Several lines of evidence point to FGSG_00080 as the *NAT1* paralog in *F*. *graminearum* that is involved in Bx metabolite detoxification. Glenn et al. [42] ran a broader comparative genomic and phylogenetic analysis of NAT proteins across kingdoms of life. Structure and homology to *NAT1* from *F*. *pseudograminearum* and *F*. *verticillioides* predicts FGSG_00080 (*FGRAMPH1_01G00243*) which is located on chromosome 1 at position 309468–310499 (-) [43]. The predicted protein homology for *NAT1* in *F*. *graminearum* is the putative gene *FGSG_00080*. Muscle alignment of FGSG_00080, *FPSE_08123* (*F*. *pseudograminearum*), *FVEG_12636* (*F*. *verticillioides*), and *FOC1_g10007925* (*F*. *oxysporum*) had a Pairwise % Positive (BLSM62) of 88.7% (S1A Fig). *F*. *graminearum* and *F*. *pseudograminearum* NAT proteins are more closely related to each other than to *F*. *verticillioides* and *F*. *oxysporum* (S1B Fig). This analysis is supported by Kettle et al. [13] in their comparison of the FDB2 loci in different *Fusaria*. In addition to the phylogenetic evidence, transcriptomic data (Jin-rong Xu, personal communication, June 2018) shows that FGSG_00080 transcription is the most responsive to infection on wheat 3 d after inoculation (see S2 Fig). Finally, the interactivity of NAT1 with other fungal proteins under DON-inducing conditions points to FGSG_00080, using the FunTAP algorithm for protein interactivity during DON production [44, R. Subramaniam, personal communication June, 2018]. Eight proteins were predicted or demonstrated though yeast two hybrid systems to interact with NAT1 during DON production, including transporter proteins, metabolitic enzymes, and *NAT2* and *NAT3* proteins in *F*. *graminearum* during DON production (Table 1). This information suggests the functionality of *NAT1* could influence DON production.

**Table 1:** Predicted interaction and experimental interactions of *NAT1 (FGSG_00080)* under toxin inducing conditions.

GENE ID	FUNTAP	PREDICTED FUNCTION	GENOMIC LOCUS
FGSG_06819	Y2H	U6 snRNA-associated Sm LSm5	FGRAMPH1_01G23259
FGSG_09400 (*NAT2*)	PI	arylamine n-acetyltransferase 2	FGRAMPH1_01G27187
FGSG_01066	Y2H	family sulfate permease	FGRAMPH1_01G02661
FGSG_08991	Y2H	unnamed protein product^f^	FGRAMPH1_01G28175
FGSG_07888 (*NAT3*)	PI	arylamine n-acetyltransferase 3	FGRAMPH1_01G25907
FGSG_02382	Y2H	related to tartrate transporter	FGRAMPH1_01G05717
FGSG_04977	Y2H	Rieske [2Fe-2S] domain protein*	FGRAMPH1_01G16785
FGSG_05922	Y2H	hypothetical protein^f^	FGRAMPH1_01G19085

*Determined by additional analysis using the OrthoMCL Ortholog groups

^f^*Fusarium* specific protein

PI = Predicted Interaction

Y2H = Yeast two hybrid screening

**Fig 1:**
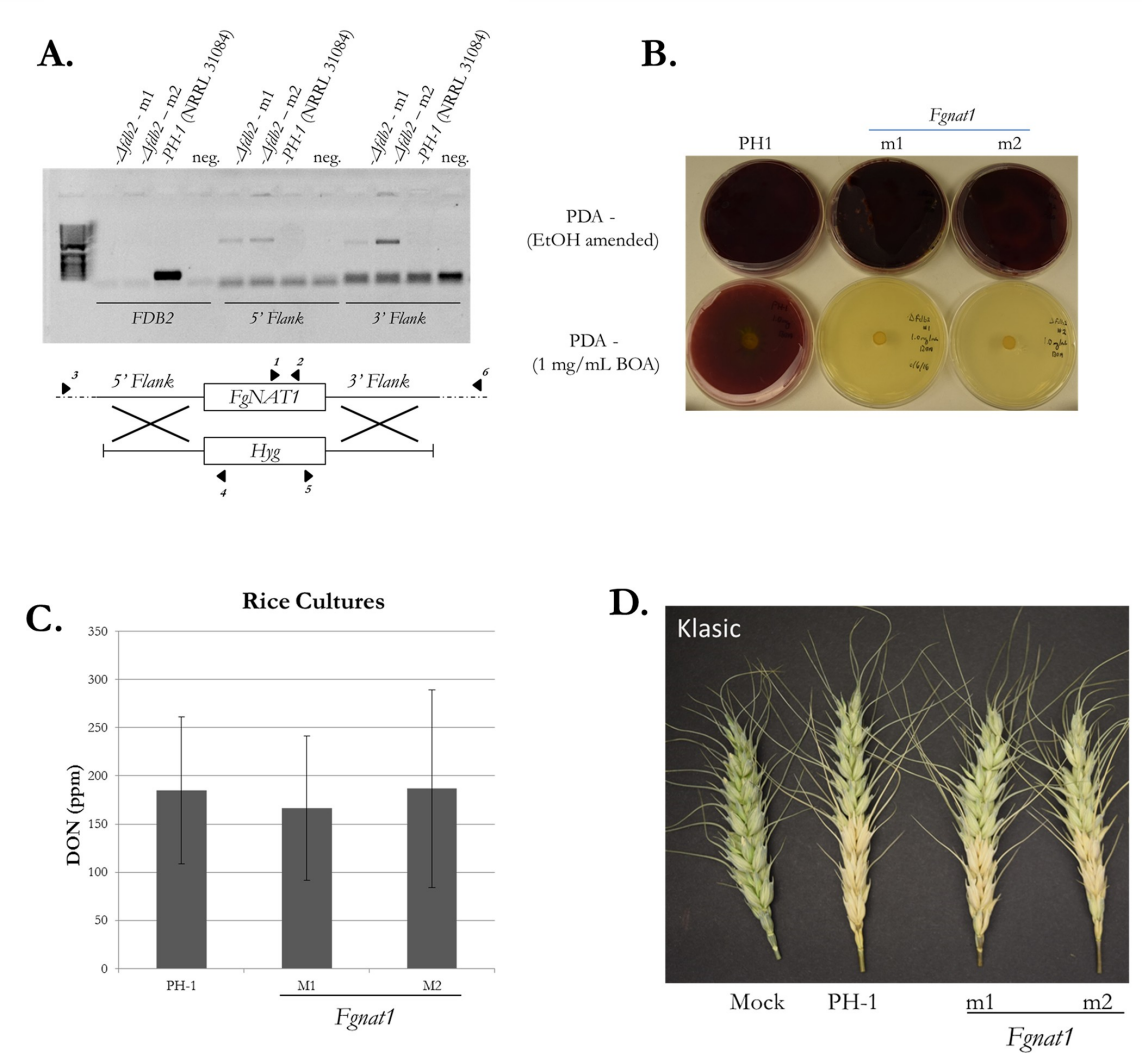
Creation and phenotyping of *Δnat1* mutants. (A) PCR of the flanks and lack of amplification of the *NAT1* locus indicated proper gene deletion in two selected mutants. *NAT1* presence, 5’ flank to hygromycin cassette, and 3’ flank to hygromycin cassette assayed, respectively, using primers 1 and 2, 3 and 4, 5 and 6. Primers are indicated by solid arrowheads. (B) PDA amended media with 1mg/mL (3 mM) of BOA completely suppressed both *Δnat1* mutants. (C) DON production between *Δnat1* mutants and PH1 in rice cultures and (D) Severity of *Δnat1* mutants and PH1 with wheat cultivar Klasic (14 dpi) inoculated 3 florets up on the spike.

Two independent *Δnat1* mutants were used for the preceding experiments. Each mutant had the correct transgenic insertion to delete *NAT1*, as verified by PCR (Fig 1A). As predicted by the function of *NAT1*, *Δnat1* mutants were unable to grow on PDA amended with 1 mg/mL BOA after 5 d compared to wild-type growth of *F*. *graminearum* PH1 (Fig 1B). However, no significant change in DON production on rice culture media in mutants vs. PH1 or in infection severity on wheat spikes was found 14 d post inoculation (dpi) (Fig 1C and 1D).

### Screening wheat cultivars for resistance against Δ*nat1* mutants

To test the effects of *NAT1* deletion on wheat infection, we compared the reaction of wheat cultivars to inoculation with the *Δnat1* mutants and PH1 strains. The wheat cultivars were chosen to represent diversity in market classes, FHB susceptibility, and genetic variability (derived from different breeding programs) (S1 Table). Treatments consisted of point inoculations of wheat spikes (mock, *Δnat1* mutants, and PH1). Analyses were conducted at 21 dpi. The reactions to the two mutants were indistinguishable (S1 File), and the subsequent presentation of *Δnat1* data is based on the means of both mutants.

**Fig 2:**
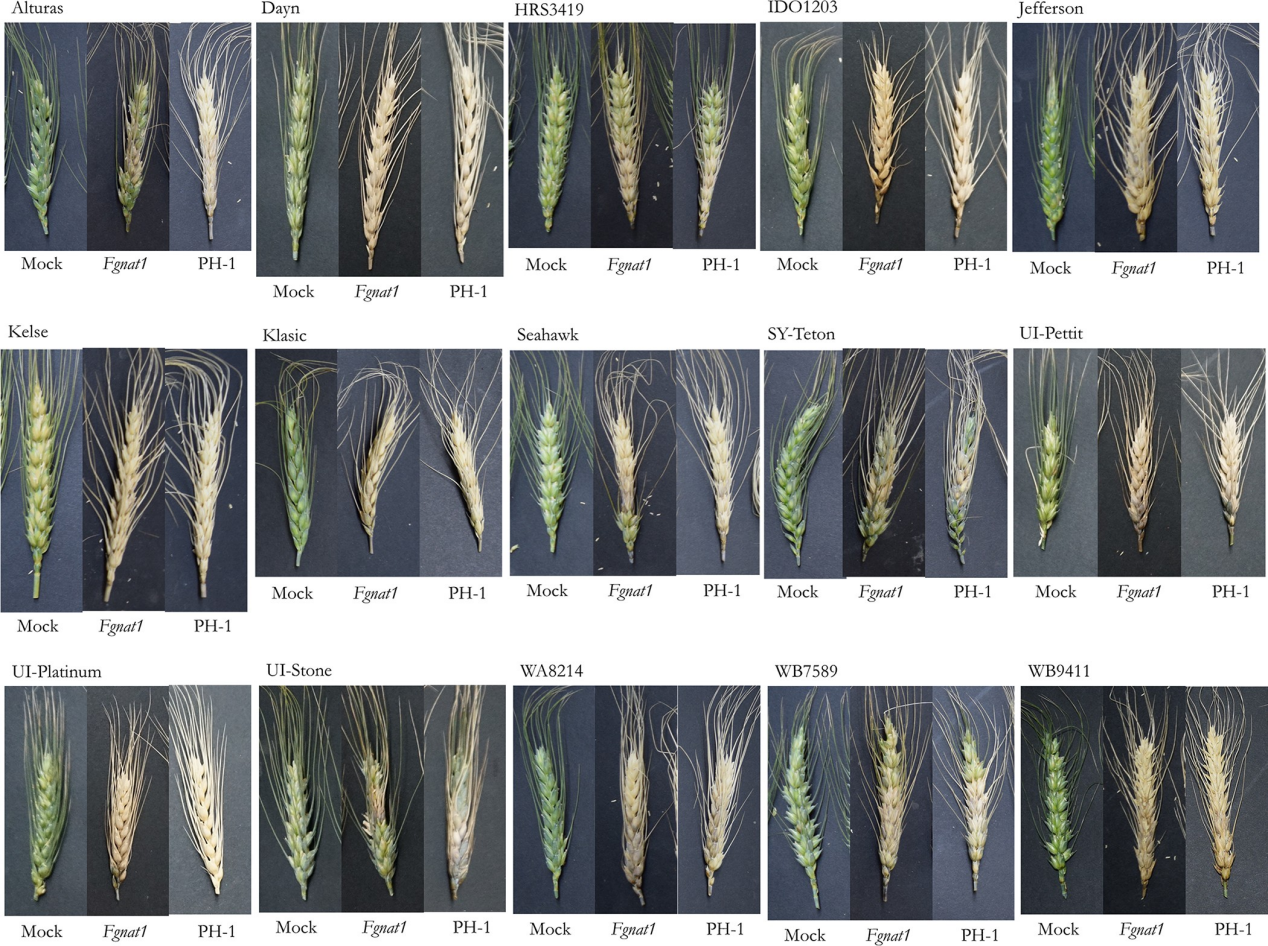
Infection symptoms of fifteen wheat cultivars with *Δnat1* mutants and PH1. Visual infection symptoms of fifteen wheat cultivars used in this study at 21 dpi. Each head is representative of a treatment (Mock, *Δnat1*, and PH1).

**Fig 3:**
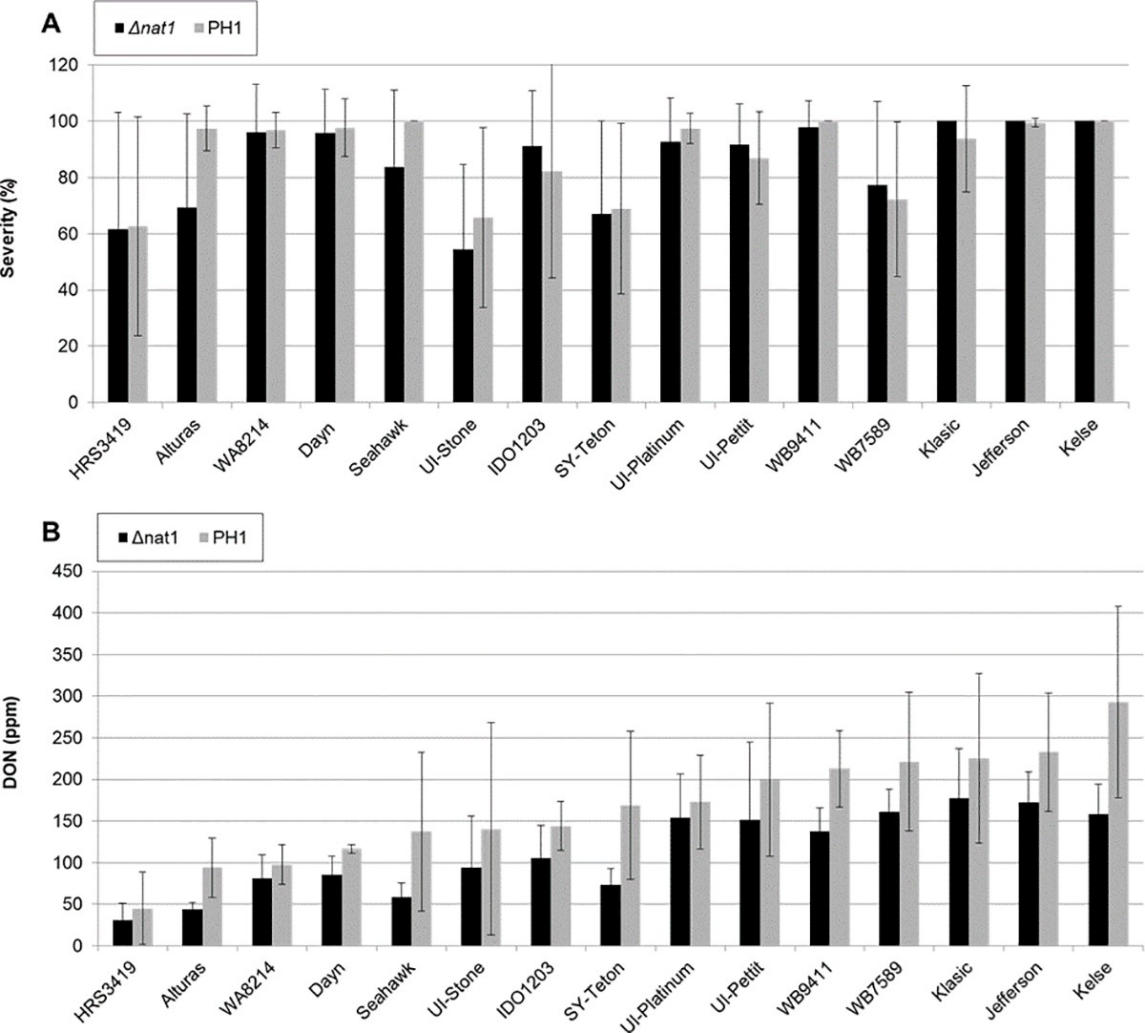
Severity and DON measurements of fifteen wheat cultivars with *Δnat1* mutants and PH1. (A) Severity and (B) deoxynivalenol (DON) measurements from fifteen cultivars, 21 dpi. Data represent 3 replicates and 4–7 heads were measured per treatment/replicate. Mock inoculations result in 0% infection severity and no DON was detected (data not shown).

Significant differences in disease severity and DON were observed among cultivars (Fig 2 and Fig 3A). DON levels were generally higher in the FHB-susceptible lines vs. the moderately resistant lines (S1 Table). None of the cultivars tested had a drastic visual reduction in symptoms 21 dpi when inoculated with *Δnat1* mutants compared to PH1 (Fig 2), nor were significant differences in severity between PH1 and *Δnat1* mutants (Fig 3A). However, differences in DON concentration between PH1 and *Δnat1* mutants were observed (Fig 3B). The wheat spikes infected with *Δnat1* mutants had significantly lower DON than PH1. HRS3419 was the most resistant cultivar tested against PH1 (severity = 62%, DON = 45 ppm) and *Δnat1* mutants (severity = 61%, DON = 30 ppm). The most susceptible cultivar tested was Kelse and severity was 100% with PH1 and *Δnat1* mutants. Kelse had the biggest reduction in DON in *Δnat1* mutants compared to PH1 on Kelse, from 292 ppm to 158 ppm. The greatest proportional differences in DON content between *Δnat1* mutants and PH1 was observed in cultivars Seahawk, Sy-Teton, Alturas, and UI-Stone with 57%, 56%, 53%, and 33% reduction in DON, respectively (Fig 3B). Dip inoculation of the barley cultivar Golden Promise was performed to check on the effects of NAT1 on a non-Bx metabolite host and no significant differences in DON or severity were found (S1 File)

### Sensitivity assays of Δ*nat1* mutants to BOA, MBOA, and DIMBOA

**Fig 4:**
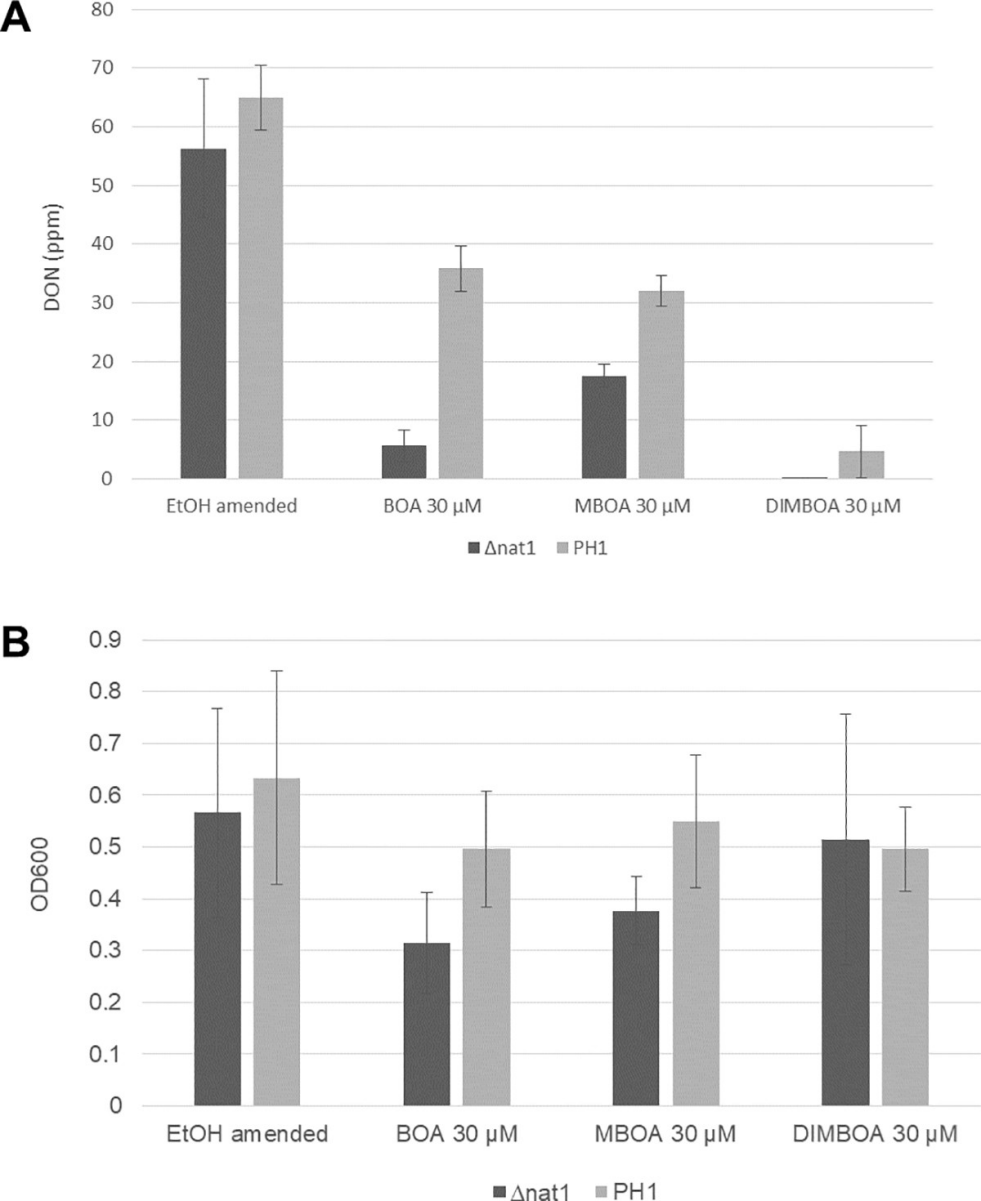
F. graminearum mutant *Δ*nat1 challenged with BOA, MBOA, and DIMBOA. (A) DON measurements from 15 mL culture (B) Corresponding OD600 reading at harvest (96 hr).

To confirm the hypothesis that the Bx metabolites BOA, MBOA, and DIMBOA affect DON production and/or growth of the fungus, PH1 and *Δnat1* mutants were exposed *in vitro* to sub-lethal levels (30 μM) for each Bx metabolite. Growth (curves at OD_600_) and DON measurements were taken at 96 h on amended TBI media (Fig 4A). Reductions in OD_600_ and DON were measured with addition of BOA, MBOA, or DIMBOA. Versus the 0 μM controls, reductions were greater for DON than for fungal growth, and the relative reductions were greater for the *Δnat1* mutants. Bx metabolites invoked differential responses. For instance, BOA caused the greatest reduction in fungal growth, but DIMBOA had the greatest reduction in DON and had the least impact on fungal growth (Fig 4B).

**Fig 5:**
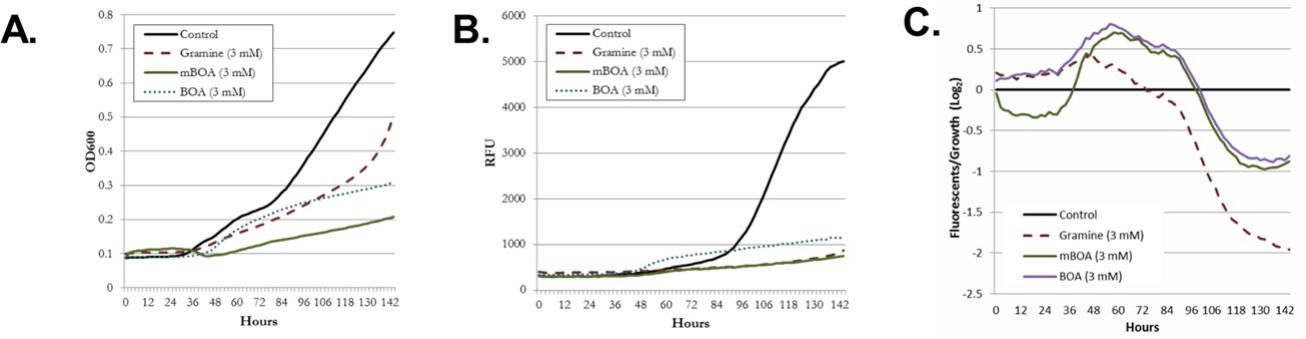
*F*. *graminearum* mutant TRI5prom::GFP responds to BOA, MBOA, and Gramine. (A) OD600 readings over 160 hr (B) Fluorescence reading for GFP (C) OD_600_ fluorescence Log_2_ ratio of readings growth suppression vs. TRI pathway suppression.

The effects of Bx metabolites on the fungus were also tested using the fluorescent strain of *F*. *graminearum*, *TRI5prom*::*GFP* [41] with fluorescent biomarkers for growth (DsRed) and expression of *TRI5* (GFP). *TRI5* encodes the trichothecene synthase responsible for DON production. This assay enable rapid assessment of conditions or compounds on fungal growth and DON production. For instance, gramine is an indole alkaloid in barley, similar to Bx metabolites in that it has been implicated in allelopathy against insects and fungi. For this reason, the sensitivity of *F*. *graminearum* to gramine was compared to to that of Bx metabolites from wheat. Exposure to 3 mM of BOA, MBOA, and gramine resulted in decreased growth (Fig 5A) and decreased GFP fluorescence (Fig 5B). The log_2_ ratio of fluorescence measurements and fungal growth (OD_600_) showed suppression of *TRI5* expression is greater than fungal growth after ~86 hours for BOA, MBOA, and gramine. These data are consistent with the data from both *in vitro* and whole-plant field studies of wheat [7, 8].

## Discussion

Allelopathic metabolites that have specific activities against pathogenic fungi could be useful as a breeding targets for control of mycotoxins in cereals. In our study we have explored the potential of Bx metabolites as a breeding target for wheat and found that deletion of a key Bx metabolite-detoxifying enzyme, *NAT1*, resulted in reduced DON on inoculated cultivars of wheat. Bx metabolites also had an effect on both wild-type and *Δnat1* mutants *in vitro*. Together, our data suggests that selection for higher levels of Bx metabolites could be effective in limiting DON in wheat for control of FHB. Alternatively, RNAi-based approaches to disrupt fungal genes important mycotoxin production and pathogenicity by host induced gene silencing (HIGS) or spray induced gene silencing (SIGS) [45–47] could be directed at targets like *NAT1*. Removing the ability for the fungus to detoxify allelopathic metabolites could increase the broad-spectrum resistance for certain cereal crops.

Deletion of *NAT1* in *F*. *graminearum* had the effect of rendering mutants unable to grow in Bx metabolite amended media, similar to the results of *NAT1* deletion in *F*. *verticillioides* [10] and *F*. *pseudograminearum* [12]. The absence of significant differences in DON production between two mutants and PH1 on rice culture media (likely devoid of Bx metabolites [17]) and on a non-Bx metabolites host (barley) demonstrated that *NAT1* is not necessary for DON production or virulence in those non-Bx metabolite environments.

The increased sensitivity to Bx metabolites of the *NAT1* mutants resulted in reduced DON when inoculated on wheat. This suggests that native levels of Bx metabolites—while insufficient to prevent FHB—do affect DON production. Consistent with this is the absence of and effect of *Δnat1* when inoculated onto barley.

*NAT1* deletion did not affect infection severity among fifteen wheat cultivars grown the Northwestern region. This is consistent with results from *F*. *verticillioides Δnat1* mutants, where maize seedling blight was unaffected [10]. Given the high expression of *NAT1* during wheat infection and the predicted interactions during DON production, deletion of *NAT1* would predict a dramatic effect on both pathogenicity and DON production, as reported for *F*. *pseudograminearum* [12]. In contrast, our study showed that *NAT1* is not necessary for either infection or mycotoxin production in the wheat cultivars tested. There may be external factors to explain the discrepancy in virulence phenotypes between the two *Fusarium* species. Lower levels of Bx compounds in the cultivars studied for this report vs that of Kettle et al. [12], or species-specific reactions with the host, may affect responses to Bx metabolites. Another possibility is that F. graminearum is suppressing Bx production during infection. Transcriptomic analysis of wheat seedlings infected with crown rot by *F*. *graminearum* revealed that expression of Bx genes 4 and 5 where being suppressed during crown rot infection [33].

Etzerodt et al. [7] had found levels of DIMBOA-Glu in to be < 20 μg/g (dry weight) in most winter and spring wheats cultivars tested. These levels obviously do not functionally protect wheat from economic damage from FHB. Increased levels of Bx metabolites could conceivably be rapidly engineered via transgenic approaches, but at present the practicality of this approach is restricted by public resistance towards bioengineering. A traditional breeding approach for selecting higher levels of Bx compounds should be explored, although considerable increases may be required. In maize, DIMBOA at levels far higher than 20 μg/g have been reported. Useful levels of resistance to the European corn borer was conferred by plants 433–704 μg/g, whereas susceptible lines had 70–91 μg/g [48].

This study shows that an RNAi-based approach to host-induced gene suppression (HIGS) of *NAT1* could moderate DON levels in the cultivars studied. The degree of DON reduction was relatively modest, but it might be useful as a component of HIGS constructs that target multiple genes. The prospect of breeding lines using traditional selection or bioengineering for higher levels of Bx metabolites is intriguing. It is well-established in the literature that wheat produces Bx metabolites, and this and other studies have implicated various Bx metabolites as antagonistic to FHB and DON production. However, knowledge is lacking of the degree of Bx metabolite variability among commercial wheat germplasm and among primary and secondary sources of variability in wheat landraces and wheat relatives. Further research on existing variability and opportunities for increase levels in wheat is justified given the potential utility of a non-transgenic genetic approach to FHB resistance.

## Supporting information

S1 Fig Muscle alignment and phylogenetic analysis of *NAT* genes. (DOCX)

S2 Fig Transcriptomic data of NAT genes FGSG_00080, FGSG_09400, and FGSG_07888 infected on wheat provided by Dr. Jin-rong Xu (Purdue, IN) in *F. graminearum* inoculated onto wheat spike at anthesis. (PDF)

S1 File Raw data of mock, two *Δnat1* mutants, and PH1 inoculated on fifteen wheat cultivars and barley cultivar golden promise. (XLSX)

S1 Table Description of fifteen cultivars from Northwestern United States. (DOCX)

S2 Table Primers used for the construction of *NAT1* deletion vector. (XLSX)
