# Supplementary material for: Deletion of the benzoxazinoid detoxification gene NAT1 in Fusarium graminearum reduces deoxynivalenol in spring wheat
Source: PLoS One. 2019 Jul 12;14(7):e0214230. doi: 10.1371/journal.pone.0214230 (PMC6625701; doi:10.1371/journal.pone.0214230)
Supplement: S1 Fig — (DOCX) [file pone.0214230.s001.docx]

**A. Muscle Alignment of NAT1 genes from *Fusaria*.** F. p. = *F. pseudograminearum*, F. g. = *F. graminearum*, F. v. = *F. verticillioides*, and F. o. = *F. oxysporum*


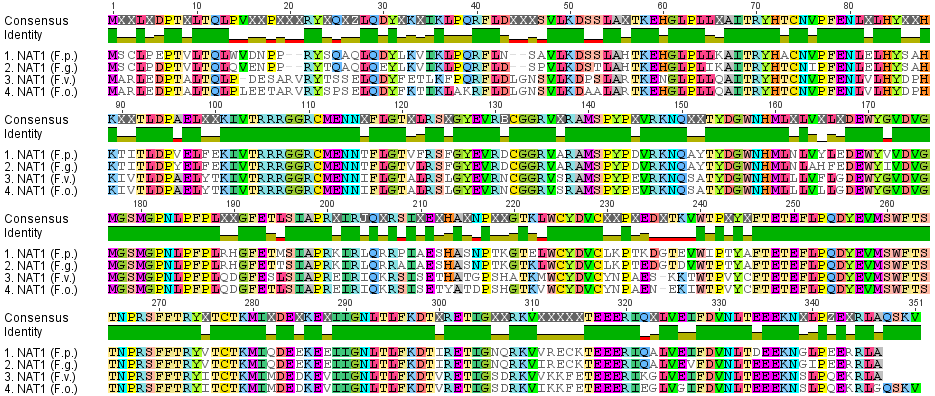


**B. Phylogenetic analysis of NAT genes in *Fusaria.*** Analysis was based on protein sequence. F. p. = *F. pseudograminearum*, F. g. = *F. graminearum*, F. v. = *F. verticillioides*, and F. o. = *F. oxysporum*.

**
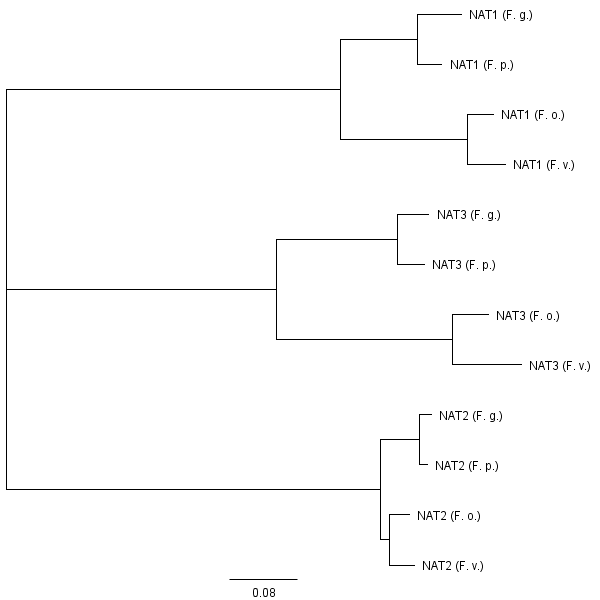
**
