# Supplementary figures and images for: Deletion of the benzoxazinoid detoxification gene NAT1 in Fusarium graminearum reduces deoxynivalenol in spring wheat
Source: PLoS One. 2019 Jul 12;14(7):e0214230. doi: 10.1371/journal.pone.0214230 (PMC6625701; doi:10.1371/journal.pone.0214230)

|            | Coni  | Hy24h | Sex8d | Inf3d | TBI3d |            | P1    | P2    | P3    | P4    | P5    | P6    | P7    | P8    |
|------------|-------|-------|-------|-------|-------|------------|-------|-------|-------|-------|-------|-------|-------|-------|
| FGSG_00080 | 1.53  | 0.07  | 2.69  | 17.89 | 1.26  | FGSG_00080 | 0.92  | 0.48  | 1.95  | 11.17 | 48.32 | 44.22 | 30.11 | 37.58 |
| FGSG_09400 | 10.42 | 22.50 | 48.40 | 45.40 | 32.37 | FGSG_09400 | 18.65 | 24.14 | 19.76 | 25.11 | 28.11 | 25.76 | 18.90 | 19.39 |
| FGSG_07888 | 2.44  | 1.00  | 1.83  | 7.90  | 1.80  | FGSG_07888 | 1.59  | 0.77  | 1.57  | 1.02  | 0.79  | 1.90  | 1.79  | 0.68  |

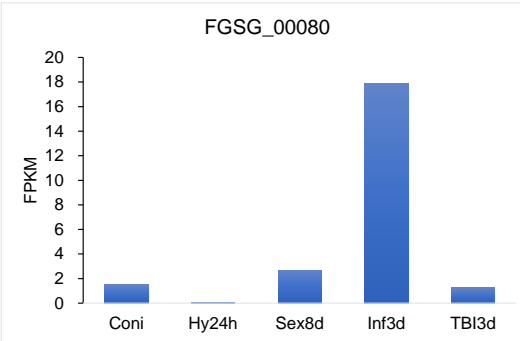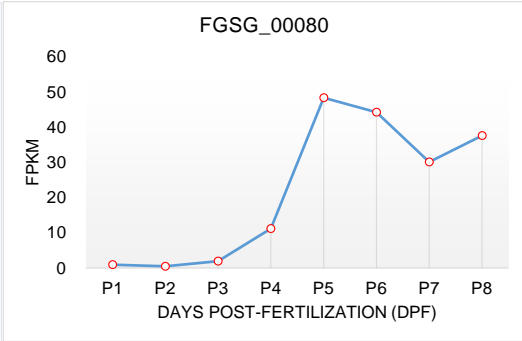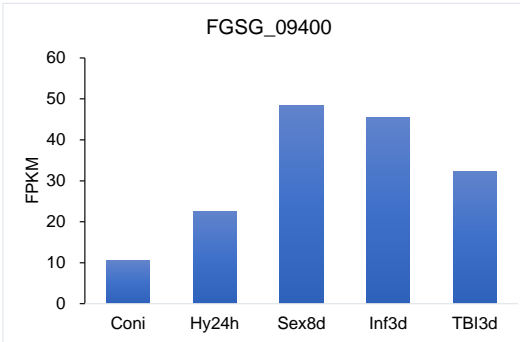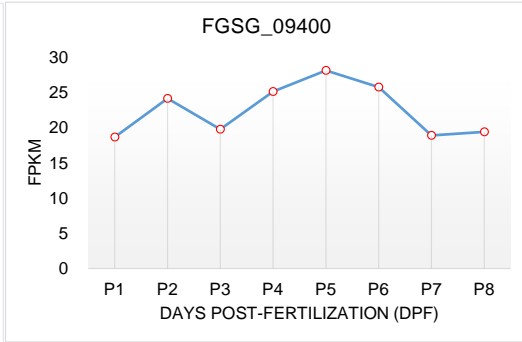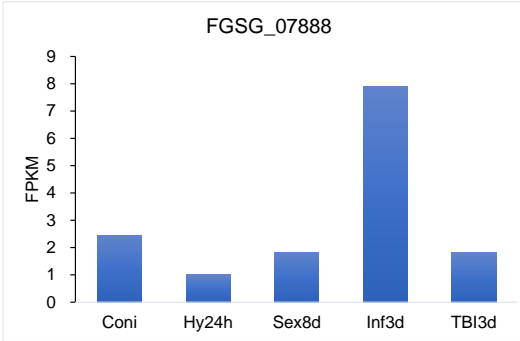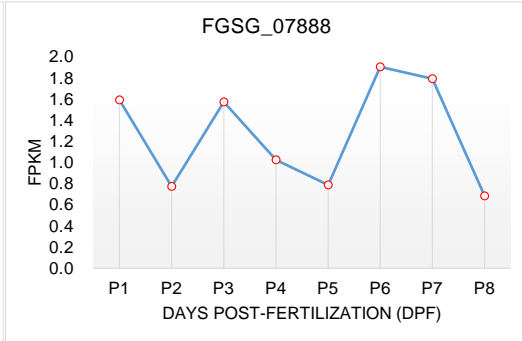

Supplement: S2 Fig — (PDF) [file pone.0214230.s002.pdf]
