# Supplementary material for: Deletion of the benzoxazinoid detoxification gene NAT1 in Fusarium graminearum reduces deoxynivalenol in spring wheat
Source: PLoS One. 2019 Jul 12;14(7):e0214230. doi: 10.1371/journal.pone.0214230 (PMC6625701; doi:10.1371/journal.pone.0214230)
Supplement: S1 Table — (DOCX) [file pone.0214230.s004.docx]

| **Wheat Cultivars** | **FHB Resistance** | **Class** | **Release Date** | **Source** | **Notes** |
| --- | --- | --- | --- | --- | --- |
| HRS3419 | Moderately Resistant | Hard Red | -- | Windfield Solutions, LLC (Land O' Lakes) |  |
| Alturas | Moderately Resistant | Soft White | 2002 | UI (Idaho AES) and USDA-ARS |  |
| WA8214 | Moderately Resistant | Soft White | 2016 | Washington AES and USDA-ARS |  |
| Dayn | Moderately Resistant | Hard White | 2012 | Washington AES and USDA-ARS |  |
| Seahawk | Moderately Resistant | Soft White | 2014 | Washington AES |  |
| UI-Stone | Moderately Resistant | Soft White | 2012 | University of Idaho |  |
| IDO1203S | Moderately Resistant | Hard White | -- | UI (Idaho AES) | IDO1203 bred to be earlier, shorter, has higher grain yield and better resistance to stripe rust |
| SY-Teton | Moderately Resistant | Hard White | 2015 | Syngenta Cereals |  |
| UI-Platinum | Moderately Susceptible | Hard White | 2014 | UI (Idaho AES) |  |
| UI-Pettit | Moderately Susceptible | Soft White | 2006 | UI (Idaho AES) |  |
| WB9411 | Moderately susceptible | Hard Red | 2014 | WestBred (Monsanto) |  |
| WB7589 | Susceptible | Hard White | 2015 | WestBred (Monsanto) |  |
| Klasic | Susceptible | Hard White | 1982 | Northrup-King |  |
| Jefferson | Susceptible | Hard Red | 1998 | UI (Idaho AES) and USDA-ARS | Jefferson wheat is a semi-dwarf hard red spring wheat intended for dry land production. |
| Kelse | Susceptible | Hard Red | 2008 | Syngenta Cereals | Kelse is resistance to the Hessian fly, adult-plant resistance (HTAP) to local races of stripe rust, and high grain protein content |
| *All cultivars are spring wheat | | | | | |

Source:
